# In response to: Defining new reference intervals for serum free light chains in individuals with chronic kidney disease: results of the iStopMM study

**DOI:** 10.1038/s41408-022-00751-0

**Published:** 2022-11-14

**Authors:** Monique C. Minnema, Joannes F. M. Jacobs

**Affiliations:** 1 Department of Hematology, University Medical Center Utrecht, Utrecht, The Netherlands; 2 Department of Laboratory Medicine, Radboud University Medical Center, Nijmegen, The Netherlands

**Keywords:** Diagnosis, Myeloma

Dear Editor,

With great interest we have read the publication of Long et al. in which they propose new reference intervals for serum free light chain (FLC) ratios in individuals with chronic kidney disease [1]. The kidney reference interval for FLC-ratio was introduced after Hutchison et al. showed that both the serum FLC concentrations and the FLC-ratio increased with worsening kidney function [2]. From the iStopMM cohort the dazzling number of 6461 participants with chronic kidney disease (CKD) without evidence of a monoclonal gammopathy were enrolled, stressing once more the importance of this large prospective population-based cohort. Long et al. now propose to further refine these reference intervals based on kidney function. As such they incorporate previous observations that both the FLC concentrations and the FLC-ratio increase with each increment in CKD stage [2, 3].

The clinical impact of these findings are substantial. It allows better identification of individuals with true monoclonal gammopathies and on the other side can avoid unnecessary referrals and unnecessary invasive diagnostics. It will also more accurately define stringent complete remission in myeloma patients with CKD who respond well to therapy. More reliable definition of the kidney reference intervals thereby contributes to accurate therapy response monitoring and allows better comparison of FLC-monitoring data between different clinical trials. Therefore, it is critical that these results can be repeated in every diagnostic laboratory worldwide and are reliable for making these important clinical decisions.

In that sense it is important to stress that the FLC measurements are obtained using Freelite reagents in a specific year on a specific analyzer. The light chain repertoire in each individual is shaped by B cell receptor gene recombination and hypermutations. This makes FLC a heterogenous group of proteins with a large charge- and size variation, that may polymerize or undergo unique post-translational modifications. As a consequence the monoclonal FLC of each individual patient is unique, which hampers exact definition of the measurand. This leaves FLC quantification subject to analytical issues such as inconsistency in linear responses, imprecision or bias due to reagent lot-to-lot variation [4]. In 2020 Rindlisbacher et al. were the first to observe a clear drift towards higher FLC-ratios over the years [5], a trend that was soon confirmed by others [6, 7]. As a consequence, in 2022 laboratories worldwide are no longer able to reproduce the defined FLC ranges in healthy controls as they were published in the seminal paper of Katzmann in 2002 [8]. The white bars in Fig. 1 illustrate that the increase in median FLC-ratios measured in large cohorts of blood bank donors is substantial. Even when measured with the same Freelite reagents measured on the same BNII nephelometer platform, a drift of more than 75% is observed [1, 7–13].

**Fig. 1:**
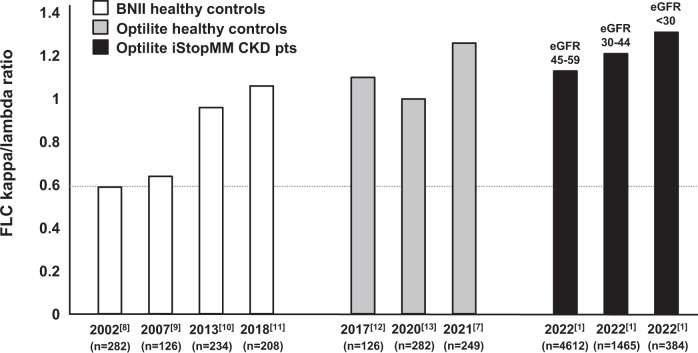
Drift in Freelite FLC-ratio observed over the years. Shown are median Freelite FLC-ratios measured with BNII (white bars) and Optilite (gray bars) on large cohorts of healthy control blood bank donors. Superimposed in this figure are the median FLC-ratios of the CKD patients published in the iStopMM study (black bars) together with the eGFR value (mL/min/1.73 m^2^). Underneath each bar the publication year plus reference, and the number of individuals per study are shown. The dotted line indicates the median FLC-ratio of 0.59 published in the landmark paper by Katzmann et al. in 2002.

To increase accessibility of Freelite testing in clinical laboratories, their use has expanded from nephelometric measurements on the BNII platform (Siemens Healthineers, Munich, Germany) to several other instrument platforms including turbidimetric methodology. Numerous studies have shown that the analytical platform affects Freelite results and warrants the introduction of instrument specific reference ranges [12]. The FLC measurements obtained by Long et al. in the iStopMM study are performed on an automated turbidimetric Optilite instrument (The Binding Site, Birmingham, England). The gray bars in Fig. 1 illustrate that various groups measured considerably higher median FLC-ratios in healthy controls on the Optilite platform compared to those measured by Katzmann in 2002 on which The Binding Site has based their FLC-ratio reference ranges (0.26–1.65). These reference ranges from 2002 were subsequently adopted in various international guidelines [14]. In Fig. 1 it is shown that the median FLC-ratios measured on the Optilite in healthy controls (gray bars) seem comparable to the median Optilite FLC-ratios in the 4612 individuals with an eGFR of 45–59 mL/min/1.73 m^2^ (first black bar) from the publication of Long et al. It is clear that the median FLC-ratio in this group (1.13) is significantly higher compared to the median FLC-ratio in blood donor controls in 2002 (0.59) and this seems to justify novel eGFR adjusted reference ranges. However, it would be interesting to know what contributes most to the increased FLC-ratio in these patients: is it the drift in FLC-ratio over time or is it indeed caused by the mild renal impairment? Analysis of age-matched FLC reference ranges in healthy controls without evidence of monoclonality retrieved from the iStopMM database could help to clarify this issue. With those data available, the outcome may be that it would be better to introduce novel Freelite reference intervals, not only for patients with impaired renal function but also for healthy controls. We would like to propose that in in case the field wants to adhere to the well-known ‘0.26–1.65’ reference ranges for scientific, clinical or standardization reasons, The Binding Site should perform a platform-wide recalibration bringing the FLC-ratio reference ranges back to 2002.

Finally, it is important to note that the eGFR adjusted reference ranges proposed by Long et al. do not apply for FLC assays from other vendors. Although overall clinical concordance appears satisfactory between the various commercially available FLC assays, significant absolute differences in FLC concentrations in individual patients can be seen, particularly at higher FLC concentrations [15]. Because of inequivalent absolute FLC values between the methods in individual patients, none of the different FLC assays can be used interchangeably. Interesting in this context is that the N Latex FLC-ratio (Siemens) is not affected by eGFR. Even in patients with severe renal failure, no adjusted reference ranges are installed for the N Latex FLC-ratio [3]. However, clinical validation of reference ranges for alternative FLC assays are warranted since international recommendations regarding clinical use of FLC measurements are based on results obtained with the Freelite test [14].

We would like to applaud Long et al. and the iStopMM initiative for their incredible important work and hope that with additional analysis the relevance for patients can be further increased.

## Data Availability

Data sharing not applicable to this article as no new datasets were generated or analyzed for this article.

## References

[1] Long TE, Indridason OS, Palsson R, Rognvaldsson S, Love TJ, Thorsteinsdottir S (2022). Defining new reference intervals for serum free light chains in individuals with chronic kidney disease: results of the iStopMM study. Blood Cancer J.

[2] Hutchison CA, Harding S, Hewins P, Mead GP, Townsend J, Bradwell AR (2008). Quantitative assessment of serum and urinary polyclonal free light chains in patients with chronic kidney disease. CJASN.

[3] Jacobs JF, Hoedemakers RM, Teunissen E, Te Velthuis H (2014). N Latex FLC serum free light-chain assays in patients with renal impairment. Clin Chem Lab Med.

[4] Jacobs JF, Tate JR, Merlini G (2016). Is accuracy of serum free light chain measurement achievable?. Clin Chem Lab Med.

[5] Rindlisbacher B, Schild C, Egger F, Bacher VU, Pabst T, Leichtle A (2020). Serum free light chain assay: shift toward a higher kappa/lambda ratio. J Appl Lab Med.

[6] Murray D, Dispenzieri A, Kumar S, Gill H, Vachon C, Snyder M (2020). Free light chain assay drift: potential for misdiagnosis?. J Appl Lab Med.

[7] Morales-Garcia LJ, Pacheco-Delgado MS (2021). Serum free light chain reference intervals in an Optilite and their influence on clinical guidelines. Clin Biochem.

[8] Katzmann JA, Clark RJ, Abraham RS, Bryant S, Lymp JF, Bradwell AR (2002). Serum reference intervals and diagnostic ranges for free kappa and free lambda immunoglobulin light chains: relative sensitivity for detection of monoclonal light chains. Clin Chem.

[9] Pattenden RJ, Rogers SY, Wenham PR (2007). Serum free light chains; the need to establish local reference intervals. Ann Clin Biochem.

[10] Campbell JP, Cobbold M, Wang Y, Goodall M, Bonney SL, Chamba A (2013). Development of a highly-sensitive multi-plex assay using monoclonal antibodies for the simultaneous measurement of kappa and lambda immunoglobulin free light chains in serum and urine. J Immunol Methods.

[11] Jacobs JFM, de Kat Angelino CM, Brouwers H, Croockewit SA, Joosten I, van der Molen RG (2018). Evaluation of a new free light chain ELISA assay: bringing coherence with electrophoretic methods. Clin Chem Lab Med.

[12] Cotten SW, Shajani-Yi Z, Cervinski MA, Voorhees T, Tuchman SA, Korpi-Steiner N (2018). Reference intervals and diagnostic ranges for serum free kappa and free lambda immunoglobulin light chains vary by instrument platform: implications for classification of patient results in a multi-center study. Clin Biochem.

[13] Bossuyt X, Poesen K, Sprangers B, Reynders M, Vercammen M, Delforge M (2020). Determination of free light chains: assay-dependent differences in interpretation. Clin Chem Lab Med.

[14] Kumar S, Paiva B, Anderson KC, Durie B, Landgren O, Moreau P (2016). International Myeloma Working Group consensus criteria for response and minimal residual disease assessment in multiple myeloma. Lancet Oncol.

[15] Fleming CKA, Swarttouw T, de Kat Angelino CM, Jacobs JFM, Russcher H (2019). Method comparison of four clinically available assays for serum free light chain analysis. Clin Chem Lab Med.

